# Metabolomic Approach to Redox and Nitrosative Reactions in Cardiovascular Diseases

**DOI:** 10.3389/fphys.2018.00672

**Published:** 2018-06-27

**Authors:** Martino Deidda, Antonio Noto, Pier P. Bassareo, Christian Cadeddu Dessalvi, Giuseppe Mercuro

**Affiliations:** Department of Medical Sciences and Public Health, University of Cagliari, Sardinia, Italy

**Keywords:** metabolomics, cardiovascular diseases, nitric oxide, reactive oxygen species, redox and nitrosative reactions

## Abstract

Metabolomics, also referred to as metabonomics, is one of the most recent innovative technologies in medicine. It offers a direct functional read-out of phenotypes by the detection, identification, and quantification of a large number of metabolites within a biological sample such as urine and blood. Metabolites (<1500 Da) represent the output of cellular metabolism, accounting for expression and activity of genes, transcripts, and proteins, and offering unique insights into small molecule regulation, which may uncover new biochemical patterns. Metabolomics research has considerable potential for translating the metabolic fingerprint into personalized therapeutic strategies. Within the field of interest, cardiovascular disease (CVD) is one of the most developed areas. However, CVD remains the leading cause of death worldwide with a marked increase in mortality rates over the past six decades. In this scenario, recent findings indicate the important role of redox and nitrosative (RN) reactions in CVD development and progression. RN reactions are generally involved in the homeostatic modulation of a wide number of cellular and organ functions. Conversely, the imbalance of these reactions may lead to a condition of allostasis that in turn can cause CVD. The aim of this review is to highlight how the use of metabolomics may be useful for the study of RN reactions related to CVD, providing a tool to understand the mechanisms underlying reactions that could lead to impaired ROS or RNS formation.

## Introduction

Cardiovascular diseases (CVDs) represent the leading cause of mortality in developed countries. Several risk factors are associated with CVDs. These are multifactorial diseases and involve a complex interplay between fixed (genotype, age, gender, and reproductive status) and modifiable (diet, smoking, exercise, and ethanol consumption) causative factors (Mozaffarian et al., 2015).

In both health and diseased states, redox and nitrosative (RN) reactions, as well as signaling, are involved in the modulation of a wide number of cellular and organ functions, such as the regulation of vascular tone by endothelial and smooth muscle cells, or the excitation-contraction coupling in cardiomyocytes (Fearon and Faux, 2009; He and Zuo, 2015).

Numerous review papers have provided overviews concerning the use of metabolomics approaches for the study of cardiac diseases, (Mercuro et al., 2011; Deidda et al., 2015a; Kordalewska and Markuszewski, 2015; Ussher et al., 2016) but none have specifically emphasized the complex area of oxidative stress. In this paper, we will review the various approaches that have employed metabolomics in order to investigate RN reactions and signaling.

## Metabolomics

Metabolomics is one of the most recent innovative technologies that aims to understand the metabolic processes within cells, tissues, organs, and organisms (Griffin et al., 2011). It is focused on the quantitative analysis of large numbers of metabolites. The latter represent the end products of genes, transcripts, and protein functions, and provide an instantaneous snapshot of biological status. While progress in genomics and proteomics analysis has allowed understanding of altered genes and proteins under a variety of perturbations, including disease conditions, metabolomics offers an alternative approach to help understand altered metabolic pathways and discover new gene functions (Griffin et al., 2011). The strong interest in metabolomics is based on the fact that even subtle changes in genes, abundance of transcripts, or levels of protein can substantially change the quantity and dynamics of metabolites. Therefore, analysis of metabolites represents a sensitive measure of biological status in health or disease (Klupczyńska et al., 2015). Altered metabolic fingerprints, which are unique to every individual, offer novel opportunities to better understand systems biology, detect or identify potential risks for various diseases, and ultimately help achieve the goal of “personalized medicine.” (Cheng et al., 2017) In this regard, a sizable number of findings have been tested for translational applications focusing on disease diagnostics ranging from early detection (Deidda et al., 2015b), to therapy prediction and prognosis,(Ussher et al., 2016) monitoring treatment and recurrence detection, as well as the important area of therapeutic target discovery (Griffin et al., 2011; Deidda et al., 2015a; Ussher et al., 2016). Moreover, current advances in analytical techniques ensure quantitation of biomarkers from even small amounts of biological samples using non-invasive or minimally invasive approaches, and facilitate high-throughput analysis required for real-time applications in clinical settings.

In this review, we focus on the application of metabolomics to the investigation of redox reactions and NO pathways, highlighting, through the presented studies, how metabolomic analysis may be applied in this research setting.

## Cardio-Metabolomics and Oxidative Stress

Among the metabolites that can be measured by metabolomics there are some that can be used to understand the mechanisms underlying reactions leading to impaired reactive oxygen species (ROS). ROS are the result of aerobic metabolism, they include the superoxide anion (O_2_^-^), hydrogen peroxide (H_2_O_2_), and hydroxyl radicals (●OH), all of which have intrinsic chemical reactive properties involving distinctive biological targets such as lipids, proteins, and DNA. Usually, ROS are correlated with oxidative stress conditions, the damaging process that involves lipids, proteins, and DNA, which suggests that ROS are the cause of several pathologies. However, it was recently demonstrated (Schieber and Chandel, 2014) that ROS also operate as signaling molecules for regulating biological processes. On the other hand, when organisms generate ROS at a greater rate than they can be balanced by the regulating systems, there is a condition of oxidative stress (Schieber and Chandel, 2014). Moreover, as previously mentioned, ROS are generated during aerobic metabolism and in particular in the trycarboxylic acid (TCA) cycle, which is known to be heavily involved in the production of metabolic intermediates and generation of reducing potential. The purpose of TCA and oxidative phosphorylation is to completely oxidize molecules of acetyl-CoA and store the chemical energy in the synthesis of molecules of ATP from ADP and phosphate. ATP is used for a variety of anabolic (energy requiring) activities of the cell. These high rate reactions can, however, also produce ROS. Another main source of ROS formation is the metabolism of purines via the xanthine oxidase pathway. In fact, ATP is rapidly depleted in hypoxia-anoxia conditions, resulting in formation of ADP, AMP, adenosine, inosine and hypoxanthine. The enzyme xanthine oxidase produces ROS as hypoxanthine is being further metabolized to uric acid in the final steps of purine degradation.

The organism compensates for ROS production with antioxidant enzymes (superoxide dismutase, catalase, and glutathione peroxidase) and intracellular molecules such as glutathione that exist in reduced (GSH) and oxidized (GSSG) forms (Lu, 2009). In addition, there are circulating antioxidants such as uric acid, ascorbate, α-tocopherol, thiols, and bilirubin.

Early pioneering studies have reported an association between cardiac diseases and the presence of abnormal metabolites related to the presence of ROS in bodily fluids (Dhalla et al., 2000). Today, current knowledge points to the important role of metabolites derived from the production of ROS and cardiac disease (**Table 1**), because these metabolites can modulate phenotypes, influencing host metabolic pathways and the immune system and determining an individual susceptibility to disease. A milestone paper was published in 2014 from Murphy’s group, which has identified how novel metabolic pathways become activated to fuel mitochondrial ROS production during ischemia-reperfusion (IR) injury (Chouchani et al., 2014). IR injury occurs when the blood supply to an organ is interrupted and then restored, and triggers many disorders, such as heart attack and stroke. To this aim, authors have used a male mouse model. Mice were allocated into four groups and exposed to 5, 30, and 30 min ischemia along with 5 min reperfusion and 30 min sham. Subsequently, LC-MS metabolomics analysis was performed on heart tissues. The resulting analysis showed that accumulation of succinate, via fumarate production and reversal of succinate dehydrogenase, is a universal metabolic signature of ischemia *in vivo*. In addition, during reperfusion, succinate dehydrogenase rapidly re-oxidized succinate in the respiratory chain thus driving mitochondrial ROS formation through RET at complex I. In conclusion, these data demonstrate that preventing succinate accumulation during ischemia is protective against IR injury *in vivo*, suggesting novel therapeutic targets for IR injury in various pathologies.

**Table 1:** Presented studies on metabolomics and ROS.

Reference	Species	Technique	Setting	Biofluid/Tissue	Discriminate Metabolites/Metabolism
Li et al., 2015	LDLR-/- mice	NMR, GC-FID/MS	Atherosclerosis	Plasma, urine	Significant drop in PUFA-to-MUFA (and PUFA-to-UFA) ratios in both the plasma and liver of WD-fed mice
	C57BL/J6 mice			Liver, Kidney and myocardial Tissue	
Chouchani et al., 2014	C57BL/6J mice	LC-MS	Ischemia-reperfusion injury	Heart	Significant increase in succinate
Lu et al., 2017	Human	QTOF/MS	Coronary Heart Disease	Plasma	glycerophospholipid metabolism including phosphatidylcholine, lysophosphatidylcholine (lysoPC), phospha- tidylethanolamine, lysophosphatidylethanolamine (lysoPE), phos- phatidylserine, lysophosphatidylserine, phosphatidylino- sitol, and lysophosphatidic acids. Among them, lysoPC (20:0), lysoPC (20:1), lysoPC (20:2), lysoPC (20:5), lysoPC (22:5), lysoPE (18:3), and glycerophosphocholine
Wang et al., 2017	Sprague-Dawley rats	UPLC-TOF/MS	Pulmonary Arterial Hypertension	Right Ventricular Tissue	Significant increase in oxidized glutathione, xanthine and uric acid and a reduction in α-tocopherol nicotinate
Varvarousis et al., 2017	Landrace/large-white pigs	NMR, LC-MS/MS	Asphyxial cardiac arrest	Plasma	Significant increase in succinate


*NMR, ^*1*^H Nuclear magnetic resonance spectroscopy; GC-FID/MS, gas chromatography with flame ionization detection – mass spectrometry; LC-MS, liquid chromatography – mass spectrometry; QTOF/MS, quadrupole time of flight mass spectrometry; UPLC-TOF/MS, ultra performance liquid chromatography – time of flight mass spectrometry; PUFA, poly-unsaturated fatty acids; MUFA, mono-unsaturated fatty acids; UFA, unsaturated fatty acids; WD, western diet; LysoPC, lysophosphatidylcholine.*

In the same year, Li and co-workers studied the origin of CVD resulting from hyperlipidemia and atherosclerosis. To this aim, The authors have analyzed metabolomic changes by means of NMR and GC-FID/MS using a well-established mouse model. This animal model is a knock-out (KO) for the low-density lipoprotein (LDL) receptor gene and is considered the preeminent animal model for experimentally investigating the pathogenesis and progression of atherosclerosis because this model can mimic the progress of hyperlipidemia-induced aortic plaques (Ishibashi et al., 1993, 1994a,b). The animals were randomly divided into three groups [control diet (CD), Western diet (WD), and diet-switched (DS)]. The CD and WD groups were fed with normal chow diet and WD, respectively, for 12 weeks, whereas the DS group was fed with WD for 6 weeks, followed by CD for a further 6 weeks. Metabolomics analyses were performed using samples of plasma, heart, kidney, liver, and urine. Among the important pathways modified by the WD diet, they found changes in energy metabolism with disruptions to glucose homeostasis including decreases in plasma glucose, renal lactate, and urinary excretion of TCA intermediates. Because of the significant presence of intermediates such as succinate, fumarate, and allantoin and the significant decrease of polyunsaturated fatty acid, it was hypothesized that the metabolic profile was associated with a condition of oxidative stress (Li et al., 2015).

Regarding succinate and fumarate, the mechanism was previously mentioned in the text (Chouchani et al., 2014); concerning allantoin and polyunsaturated fatty acid, the former was indicated by Grootveld as a product of oxidative stress resulting from oxidation of uric acid. Authors hypothesized that measurement of changes in allantoin concentration may be a useful index of free-radical reactions (Grootveld and Halliwell, 1987), whearase the latter [a reduced (PUFA/MUFA) ratio concentration] has been suggested as indicator of oxidative stress and lipid peroxidation (Galli et al., 2001).

Lu et al. (2017) performed a comprehensive metabolomics study in patients with coronary heart disease (CHD). (Lu et al., 2017) CHD is the most common type of heart disease. It is the leading cause of death in the United States in both men and women. It accounts for 30% of deaths worldwide, including 40% in high-income countries and approximately 28% in developing nations (Dalen et al., 2014; Herrington et al., 2016). CHD refers to a group of diseases that include stable angina, unstable angina, myocardial infarction, and sudden cardiac death. In order to gain a more comprehensive understanding of the pathophysiology of CHD at the molecular level, The authors have included two different groups of diseases: the stable angina (SA) and the myocardial infarction (MI) groups, which were then compared with a healthy control group (HC). All the patients recruited in this study were diagnosed and classified based on symptoms and coronary angiography (Lu et al., 2017). A plasma sample was collected from each subject and immediately frozen. Subsequently, metabolomics analysis was performed using an ultra-high performance liquid chromatography – quadrupole time-of-flight mass spectrometry (UHPLC-QTOF/MS) platform. The resulting data matrix was investigated by univariate and multivariate statistical analysis and 18, 37, and 36 metabolites were identified and recognized as being differential metabolites that distinguish SA from HC, MI from SA, and MI from HC, respectively. Among the important pathways, glycerophospholipid (GPL) metabolism emerged as the most-significantly disturbed, in particular oxidized PL on the surface of low density lipoprotein (LDL) particles and their hydrolyzed fatty acids were closely associated with CHD, however, the underlying molecular mechanisms remain poorly defined. The authors concluded that MI patients were characterized by high oxidative stress and lipid peroxidation, which is a known contributing factor in CHD.

Another interesting study was published by Wang et al., 2017 in regarding the presence of oxidative stress in right heart failure (RV) using a rat animal model (Wang et al., 2017). RV is the main cause of mortality among patients with pulmonary arterial hypertension (PAH). Recently, an experimental animal model of PAH was developed by injecting an inhibitor of VEGF receptor tyrosine kinase and ovalbumin (OVA). 2 out of 10 of animals developing PAH were dying because of RV failure within 8 weeks (Mizuno et al., 2012). Based on this experimental animal model, authors induced PAH by administering OVA and Sugen5416 to male rats and at day 50 animals were sacrificed and lung and heart tissues were collected for histological, proteomic, and metabolomics investigations. For metabolomics analysis, the collected RV tissue underwent metabolites extraction using methanol/acetonitrile followed by centrifugation. The samples were successively injected into a UHPLC-QTOF/MS system. The resulting data matrix was explored by means of partial least square-discriminant analysis (PLS-DA), which indicated the existence of a different metabolic profile between PAH RV rodents and controls. Among the discriminant metabolites, PAH RV rats had a 3.8-fold increase in xanthine and a 4.9-fold increase in uric acid, whose increased levels are index of augmented xanthine oxidase activity that is common during hypoxia and ischemia and is a well-known marker of oxidative stress. In addition, metabolomics results revealed that PAH caused a 2.1-fold increase in oxidized glutathione and a 28.2-fold reduction in α-tocopherol nicotinate, which can be because of ROS production. The authors concluded by suggesting that this model may help understanding of oxidative stress in PAH-induced RV modifications.

The idea to identify a metabolic profile in an experimental model of asphyxial cardiac arrest (ACA) compared with a model of ventricular fibrillation cardiac arrest (VFCA) was recently published by Varvarousis et al. (2017). These authors focused their attention on the metabolic differences existing between ACA and VFCA, which have not been studied yet although they represent the two most common types of CA. To this purpose, they have used landrace/large-white female pigs in which ACA and VFCA were induced. The metabolic content was characterized at different time points: at CA, during cardiopulmonary resuscitation, and in the post-resuscitation period. Plasma samples were analyzed by high resolution ^1^H-NMR spectroscopy and LC-MS/MS spectrometry in order to identify the metabolomic profiles characterizing the two pathological entities during the different phases of the experiment. Major alterations in plasma concentrations of metabolites involved the key energy production pathways. In particular, asphyxia and ventricular fibrillation differed significantly with regard to the metabolic disturbances during the peri-arrest period. Among the important metabolites the accumulation of plasma succinate was detected in the animals undergoing ACA and with worse outcome. Based on the mechanism identified by Chouchani et al., 2014, by which ROS are produced by the RET mechanism, authors concluded by suggesting a potential prognostic role for this metabolite as an indicator of ROS production and poorer outcomes.

## Cardio-Metabolomics and NO Pathways

Nitric oxide (NO) plays an important role in the development of CVD; it is a highly reactive, short-lived free radical, that exerts a wide range of biological functions, critical for the normal physiology of cardiovascular system. NO effects include cardiac contractility, regulation of vasodilation, fibrinolysis, and inhibition of several biological processes such as atherogenesis, platelet aggregation, leukocyte adhesion, and smooth muscle cell proliferation (Fearon and Faux, 2009).

In pathological states (i.e., oxidative stress) there is an impairment in NO production and its actions are consequently reduced. It is also rapidly catabolized to ONOO when it reacts with O_2_^-^.

**FIGURE 1:**
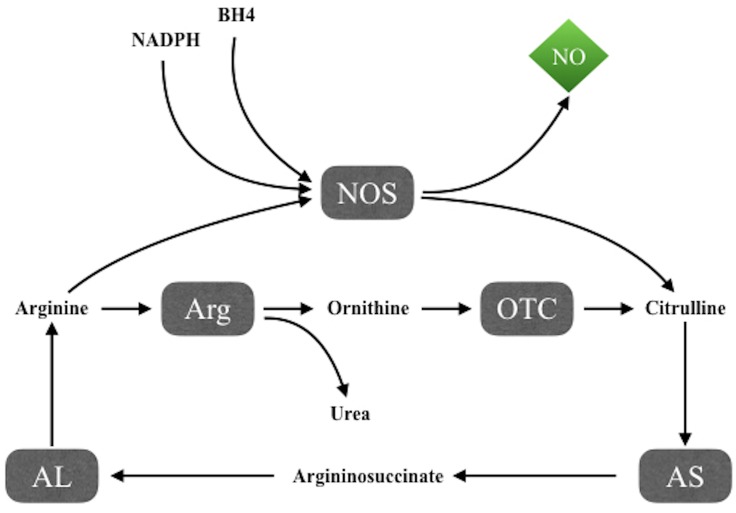
NO synthesis through the NADPH and tetrahydrobiopterin-dependent oxidation of the amino acid L-arginine to L-citrulline by NO synthases. AL, argininosuccinate lyase; Arg, arginase; AS, argininosuccinate synthetase; BH4, tetrahydrobiopterin; NOS, NO synthases; OTC, ornithine transcarbamylase.

Nitric oxide synthesis takes place through the NADPH and tetrahydrobiopterin-dependent oxidation of the amino acid L-arginine to L-citrulline (**Figure 1**), mediated by NO synthases (NOS), a family of three isoforms including two constitutive forms, neuronal NOS (nNOS) and endothelial NOS (eNOS), and an inducible isoform, iNOS. The intracellular availability of arginine is the rate-limiting step in NO production, while argininosuccinate lyase, the enzyme that produces arginine from citrulline, seems to be important both to synthesize intracellular arginine and to utilize extracellular arginine for NO synthesis (van Dyk et al., 2015).

Although NO cannot be directly measured by techniques used in metabolomics, NO-signaling in CVDs can be investigated as described in the studies presented below (**Table 2**).

**Table 2:** Presented studies on metabolomics and NO pathways.

Reference	Species	Technique	Setting	Biofluid/Tissue	Discriminate Metabolites/Metabolism
Wang et al., 2009	Human	HPLC-MS/MS	Coronary heart diseases	Plasma	Elevated levels of SDMA, ADMA and of the ArgMI index (ADMA+SDMA)/MMA)
Shao et al., 2012	Human	LC-MS/MS	Pulmonary Hypertension associated with Heart failure	Plasma	ADMA higher plasma levels and lower global arginine bioavailability ratio in acute vs. chronic heart failure.
					↑ myocardial levels of dimethylarginine dimethylaminohydrolase-1 in HF patients with sPAP > 50 mmHg
Trupp et al., 2012	Human	GC-TOF/MS	Dyslipidemia	Plasma	Cystine, urea cycle intermediates, ornithine, citrulline and lysine.
					xanthine, 2-hydroxyvaleric acid, succinic acid, stearic acid, and fructose
Zhao et al., 2015	Human	UPLC/MS/MS	Pulmonary Arterial	Lung tissue	Sphingosine-1-phosphate metabolites
	GC-MS		Hypertension		Heme metabolites
					Arginine, creatine, ornithine, and urea
Lewis et al., 2016	Human	LC-MS	Pulmonary Arterial Hypertension	Plasma	Arginine, ornithine, citrulline, ADMA, SDMA.
	C57Bl/6 mice				Indoleamine 2,3-dioxygenase (IDO)–dependent tryptophan metabolites, tricarboxylic acid intermediates, and purine metabolites.
Deidda et al., 2017	Human	NMR	Pulmonary Arterial Hypertension in systemic sclerosis	Plasma	Acetate, alanine, lactate, and lipoprotein.
					γ-aminobutyrate, arginine, betaine, choline, creatine, creatinine, glucose, glutamate, glutamine, glycine, histidine, phenylalanine, and tyrosine
Kirov et al., 2017	Human	GC-MS	Coronary artery diseases (CABG)	Arterial and Coronary sinus plasma	long-chain acylcarnitines, arginine, short-chain acylcarnitines and glycerophospholipids


*HPLC-MS/MS, high performance liquid chromatography – tandem mass spectrometry; LC-MS/MS, liquid chromatography – tandem mass spectrometry; GC-TOF/MS, gas chromatography – time of flight mass spectrometry; UPLC-TOF/MS, ultra performance liquid chromatography – tandem mass spectrometry; GC-MS, gas chromatography – mass spectrometry; LC-MS, liquid chromatography – mass spectrometry; NMR, ^*1*^H Nuclear magnetic resonance spectroscopy; SDMA, symmetrical dimethylarginine; ADMA, asymmetrical dimethylarginine; MMA, N-mono-methylarginine; ArgMI index, integrated index of arginine methylation; HF, heart failure; sPAP, systolic pulmonary artery pressure.*

Wang et al. (2009) evaluated the relationship between post-translational modification products of arginine methylation and CHD, concluding that “factors beyond direct NOS inhibition contribute to the clinical associations between methylarginines and CAD outcomes.” (Wang et al., 2009) They performed a metabolomic study analyzing 1011 plasma samples from subjects undergoing elective diagnostic cardiac catheterization using stable isotope dilution HPLC-MS/MS in order to evaluate the association of specific metabolites with future major adverse cardiac events (MACE: myocardial infarction, stroke, and death). Patients were followed-up for 3 years. They evaluated plasma levels of asymmetrical dimethylarginine (ADMA, endogenous NOS inhibitor), symmetrical dimethylarginine (SDMA, which lacks NOS inhibitory activity), N-mono-methylarginine (MMA, a potent NOS inhibitor), methyl-lysine (Methyl-Lys, an unrelated methylated amino acid), arginine, and its major catabolites (citrulline and ornithine).

An augmented prevalence of obstructive CAD was observed in patients with higher levels of SDMA and lower abundance of MMA, whereas ADMA was not correlated. On the other hand, elevated levels of both SDMA and ADMA, as well as an integrated index of arginine methylation [ArgMI(ADMASDMA)/MMA] were shown to be independent predictors of MACE; of note, the latter result was predictive of MACE even after adjustments for global arginine bioavailability (Wang et al., 2009).

The authors suggested that ArgMI and the global arginine bioavailability ratio (GABR, an integrated index of arginine bioavailability), seems to be associated with different aspects of cardiovascular risk. In fact, ArgMI seems to have a superior prognostic utility when evaluated in the setting of secondary prevention, probably because of the association between later stages of atherosclerotic progression and pathways involving protein arginine methylation, proteolysis, and impairment in NO production. On the contrary, the capacity of GABR to enhance prognostic value in primary prevention could be related to a deeper link between the NO precursor arginine bioavailability and plaque initiation and development (Wang et al., 2009).

Various studies showed elevated levels of ADMA in both chronic (Usui et al., 1998) and acute heart failure (Mullens et al., 2003; Dückelmann et al., 2007; Tang et al., 2008). However, the mechanisms underlying this dysregulation in arginine metabolism in human heart failure are generally unknown.

In a study published in 2012 on Journal of American College of Cardiology (Shao et al., 2012), 68 patients affected by advanced decompensated heart failure and 57 subjects with stable chronic heart failure were enrolled. The authors measured ADMA plasma levels and the GABR (arginine/ornithine citrulline) using tandem mass spectrometry.

Results showed higher plasma ADMA levels and lower GABR in advanced decompensated heart failure subjects in comparison with values observed in chronic heart failure patients, and these features were associated with both higher systolic pulmonary artery and central venous pressures.

A relative deficiency of NO could result as a consequence of both reduced substrate (L- arginine) bioavailability and/or impaired production due to inhibition by ADMA and could contribute to disease progression, explaining, at least in part, a more favorable long-term outcome observed in advanced systolic heart failure subjects treated with drugs that release NO (Mullens et al., 2008, 2009).

On the other hand, The authors found increased myocardial levels of dimethylarginine dimethylaminohydrolase-1 in chronic heart failure patients with systolic pulmonary artery pressure (sPAP) >50 mmHg and diminished levels in those with sPAP <50 mmHg. Overall, the data of Shao et al. (2012) seem to suggest that impaired NO synthesis could lead to both an endothelial and myocardial dysfunction thus determining an increased in sPAP.

Trupp et al. (2012) performed a gas chromatography-time-of-flight mass-spectrometry-based metabolomic study to evaluate the effect of simvastatin treatment on intermediary metabolism. The authors enrolled 148 subjects who were profiled at basal time and after 6 weeks of treatment with 40 mg/day simvastatin, then randomly selected 100 patients among the LDL-C lowering treatment responders and 24 each from both the top and bottom 10% of the response distribution, thus identifying the “good” and “poor” responders, respectively. The drug exposure metabolic fingerprint of the whole group of responders included essential amino acids, lauric acid, and alpha- tocopherol. Simvastatin efficacy in lowering LDL correlated with changes in concentration of cystine, an intermediate in the urea cycle, and the dibasic amino acids ornithine, citrulline and lysine. The latter share plasma membrane transporters with arginine, which is the rate-limiting substrate for NOS. Moreover, an orthogonal partial least square discriminant analysis (OPLS-DA) showed that xanthine, 2-hydroxyvaleric acid, succinic acid, stearic acid, and fructose were the metabolites whose basal pre-therapy levels were able to predict the good or the poor responses to simvastatin treatment (Trupp et al., 2012).

Notably, xanthine is the substrate of xanthine oxidase, whose enzymatic activity, producing H_2_O_2_, is implicated in ROS generation. Because ROS are able to uncouple NOS activity, a lower degree of xanthine and purine catabolism may lead to more intense NOS signaling. In line with these findings, hyperuricemia, which could result from increased purine degradation, is able to determine impaired NO production by inhibiting arginine transport at the level of NOS associated CAT-1 transporter (Trupp et al., 2012).

Pulmonary arterial hypertension is a vascular disease characterized by persistent pre-capillary pulmonary hypertension (PH), which can lead to premature death, often because of RV. In a study conducted on lung tissue of patients with PAH (*n* = 8) and controls (*n* = 8), Zhao et al. (2015) carried out a high-throughput liquid-and-gas-chromatography-mass spectrometry based metabolomic analysis, showing a genetic profile characterized by increases in both sphingosine-1-phosphate and heme metabolites, and a derangement of arginine/NO pathways, the latter represented by a decrease of arginine and an increase of creatine, ornithine, and urea (intermediates following arginine in the pathway) in PAH lung tissue compared to normal lung. The authors’ findings showed that in PAH, expression of the gene Arginase-1 (*ARG1*) was significantly increased whereas that of NOS-1 (*NOS1*) was decreased and this phenomenon may be the result of feedback mechanisms due to disrupted arginine metabolism with excessive intracellular and extracellular NO levels. Furthermore, the latter, reacting with ROS, may lead to peroxynitrite production, thus exacerbating cellular damage (Zhao et al., 2015).

Lewis et al. (2016) conducted a metabolomic study to verify whether it might be possible to identify a metabolic profile of right ventricular-pulmonary vascular (RV-PV) dysfunction.

They enrolled 71 individuals who represented the discovery cohort and who underwent right heart catheterization (RHC) and radionuclide ventriculography at rest and during exercise. Subsequently, their plasma samples were analyzed using a GC-MS-based metabolomic technique. The authors analyzed plasma concentrations of 105 metabolites, validating the results in a second group that underwent invasive hemodynamic evaluations and in an independent cohort with or without PAH.

The authors identified a new association between RV-PV dysfunction and circulating indoleamine 2,3-dioxygenase (IDO)–dependent tryptophan metabolites (TMs), tricarboxylic acid intermediates, and purine metabolites; moreover, they confirmed previously described associations with arginine–NO metabolic pathway constituents. In fact, levels of arginine-NO metabolites (arginine, ornithine, citrulline, asymmetric dimethylarginine, and symmetric dimethylarginine) were related to RV-PV dysfunction indexes. The ratio of arginine to ornithine + citrulline, which previously emerged as an index of arginine bioavailability and a potential biomarker of specific forms of PH, was shown to be inversely related to pulmonary arterial pressure, pulmonary vascular resistance (PVR) and to changes in mean pulmonary arterial pressure relative to change in cardiac output; the latter seems to suggest that this ratio could indicate impaired NO-mediated vasodilation (Lewis et al., 2016).

In 2017, our group performed a metabolomics study on patients affected by systemic sclerosis (SSc) and who were free of pulmonary fibrosis (Deidda et al., 2017). All subjects underwent a RHC, during which a blood sample was collected at the level of the distal peripheral circulation of the pulmonary arteries. On the basis of values of PVR, we divided the population into 2 groups: A (PVR = 1.16 ± 0.23 WU) and B (PVR = 2.67 ± 0.67 WU; *p* < 0.001 vs. Group A). We applied an Orthogonal Signal Correction–PLS–DA to metabolomics data, obtaining clear clustering; SSc patients with PAH showed increases in acetate, alanine, lactate, and lipoprotein levels and decreases in γ-aminobutyrate, arginine, betaine, choline, creatine, creatinine, glucose, glutamate, glutamine, glycine, histidine, phenylalanine, and tyrosine levels.

Of relevance to this paper, and of particular interest, decreased levels of arginine, glutamine and glutamate were found in SSc patients with higher PVR and PAH (Group B).

In fact, arginine depletion has been correlated with pulmonary arterial vasoconstriction (Watts et al., 2012). Moreover, arginine deficiency can result in various important disease states, i.e., NO production preservation and reduction in superoxide anion generation.

On the other hand, glutamine and glutamate modulate NO production and the elastic response of the thoracic aortic wall (Schachter, 2007).

An interesting heart surgery metabolomic study was conducted in the setting of coronary artery bypass surgery, evaluating the differences between the off-pump and on-pump techniques. Twenty consecutive patients undergoing Coronary Artery Bypass Graft surgery (CABG) (10 operated off-pump and 10 on-pump) were enrolled in the study; blood samples obtained from paired arterial and coronary sinus were analyzed using a MS technique. Levels of thirteen metabolites were altered by the surgery; in detail, off-pump patients showed higher levels of long-chain acylcarnitines and lower levels of arginine, whereas those who underwent on-pump intervention had more short-chain acylcarnitines and less glycerophospholipids. Interestingly, the end-of-surgery concentration of plasma arginine, the NO precursor, showed an inverse correlation with amounts of post-operative vasopressor needed in the intensive care unit. In fact, arginine concentrations in off-pump patients were lower and required more vasopressor therapy, contrary to on-pump ones (Kirov et al., 2017).

Previous research (Czerny et al., 2000; Dybdahl et al., 2004; Tomic et al., 2005) has already found higher IL-10 levels at the end of surgery in on-pump patients than in off-pump. Because IL-10 is able to inhibit macrophage NOS2, it is plausible that IL-10 may inhibit iNOS in on-pump CABG, thus determining a reduction in NO production and, consequently, a decrease in arginine catabolism through iNOS. The consequence is higher arginine in plasma and less vascular dilatation requiring less vasopressin from noradrenaline.

Collectively, metabolomics can be used to indirectly investigate NO-pathways through the analysis of metabolites involved in NOS activity or modulation, such as arginine, ornithine, citrulline, glutamine and glutamate, ADMA and SDMA. Furthermore, other indices such as ArgMI and GABR could be useful in this kind of investigations.

## Conclusion

The reported studies demonstrate the potential ability of metabolomics to investigate ROS and NO pathways and constitute a basis toward future and wider applications.

Metabolomic techniques have the limitation that they are not capable to directly identify ROS and NO; however, other molecules (see **Tables 1**, **2**), involved in RN reactions, can be measured and analyzed, thus allowing an indirect investigation of these pathways.

The success of this approach depends on how metabolomic data are integrated and contextualized with clinical and instrumental data, as exemplified in the presented studies. Moreover, the knowledge of biochemical pathways related to identified metabolites and of their biological role, both in physiological and pathological states, is crucial for the correct interpretation of the findings resulting from the application of metabolomics to the investigation of ROS and NO metabolism and signaling.

Finally, the association with the other “omics” sciences can improve the capacity of metabolomics to be effectively applied to this particular setting of investigations.

## Author Contributions

MD and AN performed the bibliographic research. All authors evaluated retrieved papers and their reference lists to identify additional relevant articles. MD, AN, and PB wrote the manuscript. CC and GM supervised the manuscript.

## Conflict of Interest Statement

The authors declare that the research was conducted in the absence of any commercial or financial relationships that could be construed as a potential conflict of interest.
